# Assessing possible moderators on the association between frequency of contact with non-cohabitating adult children and depressive symptoms among community-dwelling older adults

**DOI:** 10.1192/j.eurpsy.2023.517

**Published:** 2023-07-19

**Authors:** J. Noh, H. W. Roh, Y. Rho, M. Kim, J. Beon

**Affiliations:** Psychiatry; ^2^Ajou Univ. School of Med., Suwon, Korea, Republic Of

## Abstract

**Introduction:**

With the increase of older adult population, late life depression is emerging as a major problem in many countries as it significantly deteriorates function and quality of life in older adults. Late life depression is a multidimensional disease that requires intervention in biopsychosocial perspective. Recent reduction in family size and rapid simplification of generations are making this decrease more dramatic. Thus, single older households are becoming general type of household in late life, emphasizing the importance of social engagement in late life depression

**Objectives:**

the purpose of this study is to assess the correlation between the frequency of face-to-face and non-face-to-face contact with non-cohabitating adult children and late life depression after the COVID-19 pandemic. Additionally, we hypothesized 10 possible moderators and evaluated the moderation effect of each moderator on the correlation. By identifying groups of older adults that are more affected by contact with their children, the understanding of the relationship between late life depression and family contact in older adults might provide insights to set intervention targets in the community.

**Methods:**

Older adults who completed the Living Profiles of Older People Survey in Korea were included. In total, 7,573 participants were analyzed by measuring their contact frequency and depression symptoms. Regression analysis was done adjusting covariates. Process macro was used to verify the moderating effects of variables.

**Results:**

Multivariable logistic regression analysis showed that both infrequent face-to-face (OR=1.87, 95% CI=1.56-2.23) and non-face-to-face contact (OR=1.22, 95% CI=1.03-1.44) with non-cohabitating adult children group was associated with higher risk of late life depression compared to a frequent contact group. Further linear regression analysis, which viewed depressive symptoms as SGDS-K score, indicated consistent results in face-to-face and non-face-to-face contact (estimate=0.468, standard error [SE]=0.091, p<0.001 and estimate=0.262, standard error [SE]=0.079, p<0.001, respectively). Finally, using moderation analysis, association with late life depression and frequency of face-to-face contact was moderated by age, quartiles of household income, number of chronic diseases, frequency of physical activity, existence of spouse, and nutritional status (NSI) whether effect of frequency of non-face-to-face contact on late life depression was increased by participation in social activity, frequent physical activity, and good cognitive function (MMSE-DS score)(p for interaction<0.05).

**Conclusions:**

Frequent contact of non-cohabitating children lowers the risk of later life depression. Several variables were found significant in moderating contact frequency-depression symptoms.

**Disclosure of Interest:**

None Declared

